# New Insights into Epigenetic Regulation of T Cell Differentiation

**DOI:** 10.3390/cells10123459

**Published:** 2021-12-08

**Authors:** Avik Dutta, Harini Venkataganesh, Paul E. Love

**Affiliations:** 1 Section on Hematopoiesis and Lymphocyte Biology, *Eunice Kennedy Shriver*, National Institute of Child Health and Human Development, National Institutes of Health, Bethesda, MD 20892, USA; avik.dutta@nih.gov (A.D.); hvenkat1213@gmail.com (H.V.); 2 Rensselaer Polytechnic Institute, Troy, NY 12180, USA

**Keywords:** epigenetics, T cell differentiation, cancer, autoimmune disease

## Abstract

Immature CD4^−^ CD8^−^ thymocytes progress through several developmental steps in the thymus, ultimately emerging as mature CD4^+^ (helper) or CD8^+^ (cytotoxic) T cells. Activation of naïve CD4^+^ and CD8^+^ T cells in the presence of specific cytokines results in the induction of transcriptional programs that result in their differentiation into effector or memory cells and in the case of CD4^+^ T cells, the adoption of distinct T-helper fates. Previous studies have shown that histone modification and DNA methylation play important roles in each of these events. More recently, the roles of specific epigenetic regulators in T cell differentiation have been clarified. The identification of the epigenetic modifications and modifiers that control mature T cell differentiation and specification has also provided further insights into how dysregulation of these processes can lead to cancer or autoimmune diseases. In this review, we summarize recent findings that have provided new insights into epigenetic regulation of T cell differentiation in both mice and humans.

## 1. Introduction

T cell differentiation is a tightly regulated process. Recent studies have shown that epigenetics plays a significant role at all stages of the differentiation process. In this review, we will briefly introduce the different stages of T cell differentiation and will discuss recent findings on the epigenetic regulation of this process.

### 1.1. Generation of Differentiated T Cells

**Figure 1:**
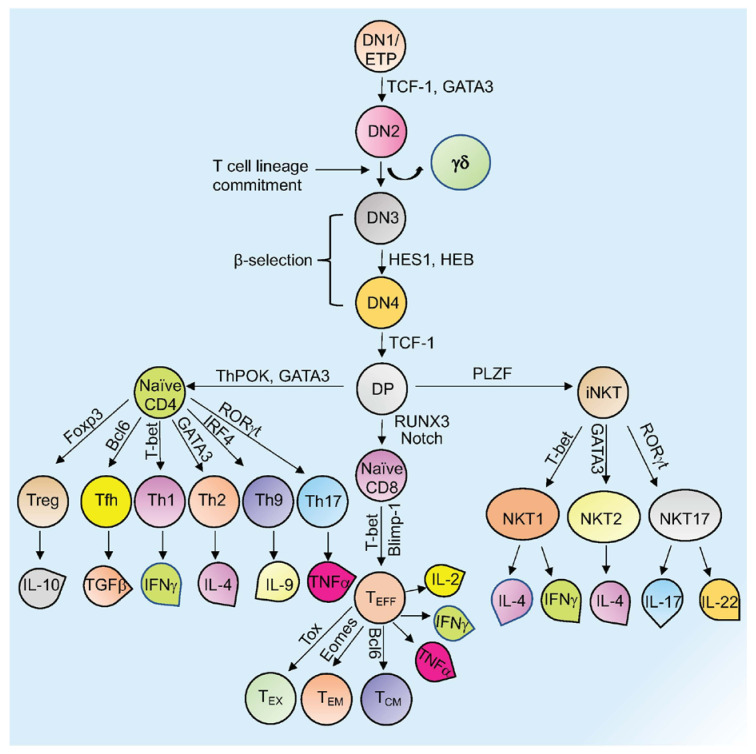
Overview of T cell development and differentiation. Schematic representation of thymopoiesis in mice. Following a series of DN (1–4) stages, DP cells develop into naïve CD4^+^, naïve CD8^+^, or natural killer T cells (NKT). Several transcription factors regulate this process. Different T cells secrete various cytokines to exert their activity. Signature transcription factors and cytokines designated to different cell types are shown. See the text for more details about each type of cell and their function (T_ex_= exhausted T cells; T_EM_ = T effector memory T cells; T_CM_= T central memory T cells; T_EFF_ = effector T cells; Th= T helper T cells; Tfh= T follicular helper T cells; Treg= regulatory T cells).

Mature T cells originate in the thymus and after going through various developmental stages they exit the thymus to circulate in the peripheral lymphoid organs including the spleen and lymph nodes [1,2]. Originating in the bone marrow or fetal liver, thymic seeding progenitor cells (TSPs) (also known as early thymic progenitor cells (ETPs) or DN1 cells (CD4^−^ CD8^−^ double negative; DN)) enter the thymus, where they progress through four DN stages (DN1-4) (Figure 1) [3]. DN3 cells expressing the pre-TCR (pre-T cell receptor) transit to the DP (CD4^+^ CD8^+^, double positive; DP) stage (Figure 1) [3,4]. In the cortex, DP thymocytes undergo a process of positive and negative selection which is determined by TCR signaling in response to binding to self-peptide-self-major histocompatibility complexes (self-pMHC) and then commit to either the CD4^+^ or CD8^+^ SP (single positive; SP) lineage (Figure 1) [2,3,4,5]. Immature SP thymocytes undergo further differentiation to generate either MHC Class II restricted CD4 SP helper or MHC Class I restricted CD8 SP cytotoxic lineage cells [3,4].

Other subsets of T cells generated in the thymus include regulatory T cells (Tregs) and natural killer T cells (NKT; Figure 1). Naïve human CD4^+^ T cells, which are now considered to be a heterogenous population, can be subcategorized based on CD31 (also known as PECAM1) expression, into the youngest (recent thymus emigrant) naïve T cells (CD31^+^) and more established “mature” naïve T cells (CD31^−^) [6,7]. After encountering antigen, naïve CD4^+^ T cells differentiate into CD4^+^ effector T cells, which can take the form of well-defined subgroups that express distinct cytokine profiles: T helper 1 (Th-1), Th-2, Th-9, T follicular helper (Tfh) cells, and Th-17 cells (Figure 1) [8]. These differentiated subgroups are not necessarily definitive as some degree of plasticity can be observed, particularly if epigenetic control mechanisms are dysregulated (discussed below). Tregs, which comprise a separate CD4 lineage, work to prevent autoimmune diseases through suppressing the immune system’s responses to self-antigens [9]. These cells express the master regulator transcription factor (TFs) Foxp3. Tregs are subdivided based on their location and mode of generation, which includes thymus-derived Tregs (tTregs), naturally occurring Tregs (nTregs), induced Tregs (iTregs), and peripherally induced Tregs (pTregs) [10,11,12,13,14,15].

CD8^+^ T cells that encounter antigen differentiate into effector T cells (T_EFF_ or Cytotoxic T cells; CTLs) that produce cytokines and cytotoxic enzymes (perforin, granzyme B) that eliminate pathogens or target cells (including pathogen-infected host cells and tumor cells) and can develop into memory T cells (Figure 1) [16]. Memory T cells (which are generated from activated CD4^+^ and CD8^+^ T cells) persevere following the generation of a primary immune response and are able to mount an enhanced secondary response to the same antigen [16]. Memory T cells can be subdivided into effector memory T cells (T_EM_) or central memory T cells (T_CM_) based on different surface markers (Figure 1) [16]. Another type of T cell with limited effector functions is known as the exhausted T cell (T_ex_). Both CD4^+^ and CD8^+^ T cells can differentiate into T_ex_ cells when encountering chronic infections and cancer (discussed in Section 3) (Figure 1) [17,18].

Natural killer T (NKT) cells are specialized TCR-positive cells with similarities to both innate NK cells and ‘conventional’ T cells that can secrete large amounts of cytokines [19]. CD1d-restricted NKT (iNKT) cells express an invariant TCRα chain (Vα14Jα18 in mice; Vα24Jα18 in humans) [19]. NKT cells can also be divided into three subsets (NKT1, NKT2, and NKT17) based on cytokine production (IFN-γ, IL-4, IL-17, and IL-22, respectively) and expression of the transcription factors PLZF (promyelocytic leukemia zinc finger), T-bet (T-box 21), Gata3, and RORγt (retinoic acid-related orphan receptor γt), respectively (Figure 1) [19,20].

### 1.2. Major Epigenetic Processes and Regulators

Epigenetics is defined as heritable changes in the phenotype without altering the DNA sequence [21]. Histone modifications, DNA methylation, nucleosome, and chromatin remodeling mechanisms are crucial players for epigenetic regulation that control gene expression [21,22]. Several previous reviews have discussed the different epigenetics processes in detail and the relationship between epigenetic dysregulation and cancer or autoimmunity [21,22,23,24,25,26]. Here, we will briefly introduce histone modifications and DNA methylation and then relate the findings relevant to the epigenetic control of T cell differentiation.

#### 1.2.1. Histone Modifications

**Figure 2:**
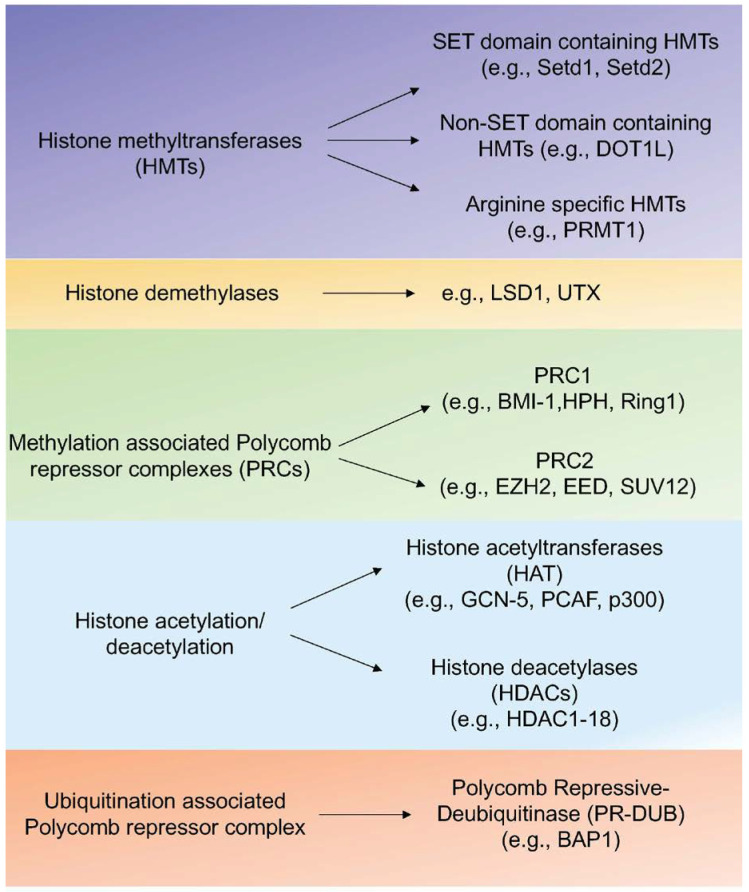
Regulators of histone modifications. Different types of regulators of histone modifications that include methyltransferases, demethylases, PRC complexes, and histone acetylation/deacetylation proteins are listed with corresponding examples.

Post-translational epigenetic modifications of histone proteins including H2A, H2B, H3, and H4 are important for the regulation of gene expression [26,27]. Methylation, acetylation, and ubiquitination are major epigenetic marks on histones. Histone methyltransferases (HMTs) catalyze the transfer of methyl groups to lysine (K= lysine) and arginine (R= arginine) residues of histone (H3 and H4) proteins [27]. Trimethylation of lysine 4 of histone H3 (H3K4), lysine 36 (H3K36), and lysine 79 (H3K79) are found at active transcription sites [28]. On the other hand, methylated H3K9, H3K27, and H4K20 are transcriptional repressive marks [29]. In Figure 2, different HMTs, polycomb group (PcG) proteins that govern the K27 methylation, and histone demethylases are listed.

Histone acetylation is another mechanism that contributes to transcriptional activation via enhanced chromatin de-condensation and DNA accessibility [30]. The acetylation status of chromatin is regulated by histone acetyltransferases (HATs) and histone deacetylases (HDACs) (Figure 2). Ubiquitination of H2A (K119) and H2B (K120) are additional epigenetic modifications of histones [31]. This epigenetic mark is associated with repressed developmental genes mediated by the polycomb group of proteins Bmi/Ring1A and is removed by the Polycomb Repressive-Deubiquitinase (PR-DUB) complex (Figure 2) [28,31].

#### 1.2.2. DNA Methylation

Maintenance of genomic stability is controlled in part by DNA methylation [23]. In mammals, DNA can be methylated by DNA methyltransferases (DNMTs) and there are enzymes that can erase pre-existing DNA methylation [24,25]. DNMTs (1–3) generate 5-methylcytosine (5 mC), which results in repression of gene expression [23]. The ten-eleven translocation protein family (TET1-3) mediates DNA demethylation, leading to activation of gene expression [24,25].

Several epigenetic regulators play pivotal roles in the differentiation of mature T cells [31,32,33,34,35]. In the following sections, we discuss the recent studies that have shed light on their contribution to mature T cell differentiation.

## 2. Epigenetic Changes during T Cell Differentiation

Epigenetic modifications greatly influence the functional differentiation of T cell subsets, including linage commitment to short-lived effectors, long-term memory T cells, T regulatory cells, and other specific T cell populations. The following sections focus on the cooperation between epigenetic changes and transcriptional programs related to different subsets of T cell differentiation. Major epigenetic regulators associated with T cell differentiation are summarized in Table 1.

### 2.1. Epigenetic Control of CD4^+^ T Cell Differentiation

Naïve T cells manifest a unique epigenetic landscape [6]. One recent study in mice found that RTEs (recent thymic emigrants) and more “mature” naïve T cells exhibit different DNA methylation patterns at key cytokine (*Il2* and *Il4*) loci, contributing to post- thymic naïve T cell maturation [68]. However, additional studies are required to fully understand the epigenetic mechanisms that regulate naïve T cell formation/maintenance and naïve T cell “maturation”. One report showed that CD4^+^ T helper (Th) cell differentiation is regulated by lysine methyltransferase (KMT) Dot1l-dependent di-methylation of lysine 79 of histone H3 (H3K79me2), which is associated with lineage-specific gene expression [36]. Loss of Dot1l (mediated by *Cd4-Cre*, which becomes active in thymocytes at the DP stage) leads to increased expression of Th-1-specific genes and overproduction of IFN-γ at the expense of Th-2 cell development, suggesting a central role for Dot1l in Th-2 cell lineage commitment and stability [36].Using *Cd4-cre*-driven conditional knockout (KO) mice, another recent report showed a contribution of Menin, a major component of the Trithorax group (TrxG) complex, in the acquisition and maintenance of Th-2 cell identity [38]. The TrxG complex catalyzes the trimethylation of H3K4, resulting in induction or maintenance of gene transcription [39]. It has been shown that Menin is an extremely specific partner for mixed lineage leukemia (MLL)1/2-containing H3K4 methyltransferase complexes [40] and that binding of the Menin/TrxG complex is required for the maintenance of Gata3 expression and Th-2 cytokine production in established Th-2 cells both in mice and humans [41,42]. Previous reports have demonstrated that Gata3 is critical for commitment of DP thymocytes to the CD4 SP lineage as well as CD4 Th-2 lineage generation [69,70]. To promote the differentiation of Th-2 cells, Gata3 directly binds to the promoters of the *Il3* and *Il5* genes, which allows for Th-2 cytokines to be expressed [71]. This study found that Menin supports memory Th-2 (mTh-2) (a subset of Th-2 cells that produce large amounts of IL-4 and IL-13 in response to antigenic re-stimulation) cell function and deletion of Menin results in a significant reduction in the number of mTh-2 cells compared with WT mice [38]. Mechanistically, they showed that Menin deficiency leads to a decrease in Gata3 expression due to reduced levels of H3K9ac and H3K4me3 at the upstream regions of the Gata3 proximal promoter [38]. Another recent report showed that Cxxc1, which can also be a subunit of the Trithorax complex, regulates the generation of functional Th-1/Th-2 cells since Cxxc1 epigenetically represses transcription of genes, including *Trib3* and *Klf2*, that are required for these differentiation processes via binding to their promoter regions [43]. The authors showed that Cxxc1 depletion by tamoxifen inducible *ER-Cre* resulted in the hyperactivation of master transcription factors including T-bet in Th-1 cells and Gata3 in Th-2 cells and hyperproduction of Th-1 and Th-2 cytokines. They also showed that Cxxc1 indirectly controls the expression of Gata3 through direct binding and regulation of *Klf2*, which negatively regulates Gata3 expression [43]. By performing ChIP sequencing, they found that Cxxc1 directly binds to the *Klf2* promoter region and that depletion of Cxxc1 resulted in a decrease in H3K4me3 levels at the *Klf2* promoter [43]. These data suggest that although part of the Trithorax complex, different components have distinct functions in the differentiation and regulation of CD4^+^ Th-1 and Th-2 cells. Interestingly, another study using *dLck-Cre* showed that T cell-specific ablation of *Cxxc1* results in the generation of severely defective Th-17 cells [44]. Mechanistically, they found that depletion of Cxxc1 results in decreased IL-6Rα expression since Cxxc1 maintains H3K4me3 modification at its promoter [44]. Depletion of IL-6Rα disrupts IL-6/STAT3 signaling, which is known to initiate Th-17 cell differentiation, resulting in the production of Treg cells instead of Th-17 cells, suggesting that Cxxc1 keeps a balance between these two lineages [44,45,46]. The contradiction between the two reports, i.e., Th-1 and Th-2 production [43] vs. Th-17 cells [44] generation due to *Cxxc1* ablation, might be due to the Cre system (*ER-Cre* vs. *dLck-Cre*) that was used as the former targets mature T cells whereas the latter acts on immature pre-selection thymocytes. Another study tested the effects of a new BET (bromodomain and extra-terminal) inhibitor, OTX015, on CD4^+^ Th-17 cells [72]. BET proteins are epigenetic regulators that recognize and bind to acetylated histones in chromatin and that regulate gene expression. The authors showed that OTX015 has anti-inflammatory effects via suppression of CD4^+^ T cell proliferation in both mice and humans and suppresses the production of Th-17 cytokines (IL-17 in particular) in humans [72]. These data suggest that BET plays an important role in CD4^+^ Th-17 cell generation and persistence. Another report identified ATF7ip (activating transcription factor 7 interacting protein, also known as MCAF1 or mAM), an epigenetic regulator responsible for repressive H3K9me3 marks via its histone methyltransferase-binding partners SETDB1/ESET [47], as a crucial regulator of Th-17 differentiation [48]. Depletion of Atf7ip (*Cd4-Cre*) resulted in impaired Th-17 differentiation and enhanced production of IL-2 in response to T cell receptor (TCR) stimulation [48]. They further showed that ATF7ip acts as an IL-2 inhibitor via suppression of *Il2* gene expression through H3K9me3 deposition in the *Il2-Il21* intergenic region [48]. Future studies will determine if ATF7ip inhibition could be useful for the treatment of Th-17-mediated autoimmune diseases. Using a combined chemico-genetic approach, another recent study showed that the histone H3K27 demethylases KDM6A (UTX) and KDM6B (JMJD3) function as central regulators of human Th subsets [49]. In this study, the authors utilized the pan-KDM6 inhibitor GSK-J4, which increases H3K27me3 decoration genome wide and suppresses the expression of RORγt during Th-17 differentiation in human CD4^+^ T cells [49]. They showed that KDM6 inhibition in mature Th-17 cells leads to reduced mitochondrial biogenesis, resulting in metabolic reprogramming and reduced expression of key metabolic TFs, such as PPRC1, which ultimately showed anti-inflammatory effects [49].

Another study also showed the importance of epigenetic control of metabolism in follicular helper T (Tfh) cell differentiation [73]. This report showed that the E3 ubiquitin ligase, Von Hippel–Lindau (VHL), was indispensable for Tfh cell development and function [73]. Using VHL conditional knockout mice (*Cd4-Cre*) and acute virus infection or antigen immunization, the authors documented that VHL positively regulates Tfh cell development and function from the very initiation stages [73]. Mechanistically, they found that VHL acts through the HIF-1α (hypoxia-inducible factor 1α)-dependent glycolysis pathway to promote the development of Tfh cells. VHL depletion leads to enhanced glycolytic activity via GAPDH, which reduces ICOS expression, a critical molecule for Tfh development, through epigenetic regulation of N6-methyladenosine (m6A) in Tfh cells [73]. Thus, this study points out the interplay between metabolism and epigenetic control on T cell differentiation [73].

Other than DNA demethylation or histone lysine methylation, histone acetylation/deacetylation also plays a major role in CD4^+^ cell fate determination [53]. Using *Cd4-cre*-mediated *Hdac3* conditional knockout in mice, one study showed that compared to wild-type mice, the peripheral numbers of CD4^+^ and CD8^+^ T cells are significantly reduced as they enter the long-lived naïve T cell pool, suggesting a block at the RTE (recent thymic emigrant) stage of T cell maturation [53]. A previous study showed that *Cd4-Cre*-mediated *Hdac3* conditional knockout mice have normal development of conventional T cells but exhibit a block in iNKT cell development [54]. Here, the authors further showed that *Hdac3*-deficient naïve peripheral T cells (both CD4^+^ and CD8^+^) have a defect in functional maturation, since these cells fail to produce TNF upon TCR/CD28 stimulation, suggesting that Hdac3 is critical for functional T cell maturation [53].

Interestingly, another study showed that the transcription factors Tcf-1 (Tcf7) and Lef-1 have intrinsic HDAC activity and are essential for repressing CD4^+^ lineage-associated genes, including *Cd4, Foxp3*, and *Rorc*, in CD8^+^ T cells via histone deacetylation [56]. *Tcf1*- and *Lef1*-deficient CD8^+^ T cells have increased H3K27Ac and H3K9Ac marks due to the diminished intrinsic HDAC activity of Tcf-1- and Lef-1 and Tcf-1- and Lef-1-deficient CD8^+^ T cells lose the ability to suppress CD4^+^ lineage-specific genes [56]. They also performed homology modeling to predict the Tcf-1 domain that would match known HDACs. They found that the Tcf-1 HDAC domain is similar in structure to a region in the Hdac8 catalytic pocket [56]. Solving the structure of Tcf-1 and Lef-1 will help understand the molecular basis of their HDAC activity. One study using histone acetyltransferase Gcn5 (encoded by *Kat2a*) conditionally deleted mice (*Lck-Cre*) revealed that Gcn5 plays pivotal roles in multiple stages of T cell development and differentiation [57]. They found a developmental block at the DN3 thymocyte stage upon loss of Gcn5 expression [57]. The authors mainly focused on T cell activation and differentiation; therefore, further studies are needed to address the mechanistic role of Gcn5 in β-selection and T cell development. However, they showed that in vitro deletion of *Kat2a* by tamoxifen treatment of naïve CD4^+^ T cells (using *ESR-Cre*) resulted in a severe defect in CD4^+^ T cell proliferation [57]. Depletion of Gcn5 also resulted in impaired IL-2 production and perturbed differentiation of Th-1/Th-17 cells but not Th-2 and Treg cell differentiation [57]. Mechanistically, they found that Gcn5 acetylates histone H3K9 to promote IL-2 production and suggested that Gcn5 may be a target for the treatment of autoimmune diseases [57].

### 2.2. Epigenetics Associated with Treg Development and Maintenance

Regulatory T cells (Tregs) are vital for the maintenance of immune homeostasis and self-tolerance [11,14,74]. Multiple studies have helped to elucidate the dynamic role of epigenetics in Treg cell development and maintenance [11,75,76,77]. One report showed that in humans, Treg commitment begins at the CD4^+^ CD8^+^ DP stage, and that Treg precursor cells have a fully demethylated FOXP3 enhancer, which leads to stable FOXP3 expression [78]. Another study showed that treatment with the DNA methyltransferase inhibitor 5-azacytidine (5-Aza) promotes Treg cell signature gene expression, including *FOXP3*, *CD25, GITR*, and *CTLA-4*, in CD4^+^ CD25^hi^ Treg precursor cells and induces development of Treg cells [79]. They further showed that 5-Aza treatment resulted in the production of increased levels of IL-2, which was indispensable for maintenance of *FOXP3* expression [79]. A recent study showed that Foxp3^+^ cell-restricted (Foxp3^YFP-Cre^) depletion of the epigenetic regulator Uhrf1 (also known as Np95 in mice and ICBP90 in humans) in mice resulted in a widespread decrease in CpG methylation, leading to de-repression of inflammatory transcriptional programs with spontaneous inflammation and destabilization of the Treg lineage [80]. Overall, this study showed that persistent DNA methylation is essential for the development and maintenance of Tregs [80]. Previously, it has been shown that DNA demethylation is needed to activate a CpG-rich *Foxp3* intronic enhancer, CNS2 (conserved noncoding sequence 2), during Treg differentiation, which leads to the formation of epigenetic memory of Foxp3 expression and protects Treg identity [81,82]. A very recent study showed that CRISPR/Cas9-KRAB (dCas9-fused to Krueppel-associated box domain) [83] mediated *Foxp3*-transcriptional silencing (using single-guide RNA specific for *Foxp3* promoter site 3) abrogates CNS2 demethylation whereas *Foxp3*-transcriptional activation promotes CNS2 demethylation [84]. This study established a causal relationship between *Foxp3* transcription and CNS2 demethylation, which may have therapeutic potential in future Treg-based therapies [84].

A recent report, utilizing a mouse model with reduced IL-2 signaling (*Il2rα*^mut/mut^), documented that reduced IL-2 signaling affects Treg cell responses in vitro and in vivo [85]. Mechanistically, they showed that IL-2 governs genome-wide chromatin accessibility in tTreg cells via regulation of the positioning of genome organizer Satb1 (Special AT-Rich Sequence Binding Protein 1) in CD4^+^ thymocytes [85]. A previous report documented that Satb1 is essential for Treg cell-lineage specification before Foxp3 expression in the thymus, but after Foxp3 expression, it is not required [86]. Here, they found that limiting IL-2 signaling leads to ectopic binding of Satb1 to ~20X more genes associated with DNA binding, immune activation, and other functions in *Il2ra*^mut/mut^ mice compared to WT thymocytes [85]. Overall, this study established a crucial role for IL-2 in generating the epigenetic identity of Treg cells prior to Foxp3 expression [85].

One recent report showed the importance of CoREST complexes that consist of the scaffolding protein Rcor1 or Rcor2, the epigenetic modifiers Hdac1 or Hdac2, and the histone H3 lysine-4 demethylase Lsd1 in the Treg suppressive function [59]. This study showed that Foxp3 is physically associated with Rcor1, Rcor2, and Lsd1 and that depletion of Rcor1 in Foxp3^+^ cells leads to enhanced expression of IL-2 and IFN-**γ,** which provides increased anti-tumor CTL responses [59]. Loss of Rcor1 in Tregs (Foxp3^YFP-Cre^) leads to diminished recruitment of Hdac1 and/or Hdac2 and Lsd1 to the promoters of the IL2 and T-bet genes and increased acetylation at histone 3 at the *Il2* promoter as well as at the promoter of T-bet (*Tbx21*), which regulates IFN-γ expression [59]. Previously, it was reported that Foxp3 inhibits the expression of these effector cytokines [12] and the current study points out that ablation of Rcor1 disrupts the CoREST complex and increases the expression of effector cytokines to target tumors [59]. Similar observations were achieved by use of the CoREST complex inhibitor Corin, suggesting possible future studies for cancer immunotherapy by targeting CoREST complexes [59,60]. One report pointed out that *in vivo* pharmacological inhibition of PRC2 complexes (GSK503) greatly diminished the severe T cell-driven autoimmunity caused by Treg cell depletion [87]. They further showed that cytoplasmic PRC2 (cPRC2) directly binds to the T cell antigen receptor subunit CD3ε and positively regulates T cell activation/signaling and suggested the development of cytoplasmic inhibitors of PRC2 will be beneficial for efficient and non-toxic immunosuppression for treatment of autoimmunity caused by excessive T cell activation [87]. Another study shed light on the role of Bmi1 in the modulation of the proinflammatory enhancer network in both mouse and human Tregs [88]. In this report, the authors showed that genetic (Foxp3^EGFP-Cre^) or transient (siRNA knockdown) *Bmi1* ablation in Tregs leads to loss of Treg identity and Tregs are converted into Th-1/Th-17-like cells due to significant upregulation of H3K27ac and chromatin accessibility [88]. This study raises concerns about the Bmi1 inhibitors that are currently under clinical trials (for diseases including glioblastoma multiforme (GBM), colorectal cancer, head neck squamous cell carcinoma) and suggests a closer look for adverse side effects [88]. Interestingly, another study points out that modulation of metabolism leads to epigenetic changes, which reprograms the differentiation of Th-17 cells towards an induced regulatory T (iTreg) fate [89]. The authors showed that a novel small molecule, (aminooxy)acetic acid, inhibits the catalytic activity of Got1 (glutamate oxaloacetate transaminase 1) responsible for increased 2-hydroxyglutarate production, which caused hypermethylation of the *Foxp3* gene locus and subsequent inhibition of *Foxp3* transcription [89]. Although the mechanism for this effect remains unclear, the results suggest that (aminooxy)acetic acid skews the differentiation towards iTreg from Th-17 cells via reduced methylation of the *Foxp3* gene locus, and increased Foxp3 expression [89]. They further showed that increased Foxp3 expression antagonizes the function of the transcription factor Rorγt (Rorc), supporting the generation of iTreg cells [89]. This study suggests that targeting the glutamate-dependent metabolic pathway may be a way to treat Th-17-mediated autoimmune diseases via keeping the balance between iTreg and Th-17 through epigenetic modifications [89].

### 2.3. Epigenetic Regulation of CD8^+^ T Cell Differentiation

CD8^+^ T cells play a critical role in removing or killing intracellular pathogen-infected cells and tumor cells. A recent study using bacterial infection as a model, characterized the epigenetic landscapes of naïve, effector, and memory CD8^+^ T cells and identified TFs that promote CD8^+^ T cell differentiation [90]. They found that the TF YY1 (yin and yang-1) promotes adoption of an effector T cell phenotype while Nr3c1 (nuclear receptor subfamily 3 group C member 1; a glucocorticoid receptor) promotes a memory-progenitor cell phenotype [90]. Age plays an important role in determining the fate of naïve T cells. One recent study showed CD8^+^ T cells from older adults have significantly reduced accessibility to genes responsible for maintaining cellular quiescence [91]. The authors further showed that compared to young adults, older adult CD8^+^ T cells have reduced expression of the TFs YY1 and NRF1 (nuclear respiratory factor 1) due to less accessibility to their chromatin binding sites [91].

The role of Ezh2, which is the catalytic subunit of the PRC2 complex that mediates di- and trimethylation of H3K27, is well established for CD8^+^ T cell differentiation and function and is reviewed elsewhere [92,93]. A new study showed that Ezh2 is required for differentiation of terminal effector CD8^+^ T cells but not for memory CD8^+^ T cell formation, although Ezh2-deficient CD8^+^ memory T cells are unable to clear infection, suggesting it is required for protective immunity [61]. Further study is needed to identify the underlying molecular mechanism behind this phenotype. As compared to the PRC2 complex, which creates repressive marks, Dot1l is associated with the positive regulation of gene transcription via mono-, di-, and trimethylation of histone H3K79 [37]. Using *Lck*-*Cre*-mediated conditional *Dot1L* knockout mice, the authors showed that loss of H3K79me2 in T cells leads to loss of naïve CD8^+^ T cells due to premature differentiation toward a memory-like state independent of antigen exposure [37]. Mechanistically, they showed Ezh2 is a target of Dot1l and loss of Dot1l affects Ezh2 function, leading to de-repression of a subset of PRC2 targets, which are actively repressed in control mice. They also showed that Dot1l ablation leads to repression of developmentally regulated genes, but the exact mechanism is yet to be determined [37]. Another study showed that CD40 ligand (CD40l), which is a member of the TNF superfamily proteins and helps in producing and maintaining cytotoxic T cells [94], is epigenetically suppressed in CD8^+^ cytotoxic T cells [95]. The promoter region of the *Cd40lg* gene was modified by methylation of CpG dinucleotides and suppressive histone lysine methylation marks. Mechanistically, they showed that enforced expression of the TF Thpok (Zbtb7b) leads to increased CD40l expression in CD8^+^ cytotoxic T cells via inhibition of Cxxc5, which interacts with the histone-lysine *N*-methyltransferase SUV39H1 to induce H3K9 methylation [95].

One recent report demonstrated that conditional deletion of *Hdac3* in CD8^+^ T cells (using CD8 lineage-specific *E8I-Cre*) leads to more cytotoxicity while total numbers of CD8^+^ T cells were unaffected, suggesting that Hdac3 acts as a negative regulator for CD8^+^ T cell cytotoxic activity [55]. They also found that Hdac3 is required for activation of CD8^+^ T cells following an acute LCMV (lymphocytic choriomeningitis virus) infection [55]. Mechanistically, they found that Hdac3 depletion leads to an increase in enrichment of H3K27ac decoration at the *Runx3* and *Gzmb* (granzyme B) genomic loci, which was essential for CD8^+^ T cell cytotoxicity [55], whereas increased H3K27ac at *Prdm1* (Blimp-1), which is a transcriptional repressor that enhances terminal differentiation of effector CD8^+^ T cells during viral infection [96], was necessary for T cell persistence during activation [55].

### 2.4. Epigenetic Directives of Memory T Cell Generation and Maintenance

Growing evidence suggests that epigenetics plays an important role in regulating memory T cell generation and maintenance. A recent report points out that CD8^+^ T cells deficient in the histone acetyltransferase CBP (Crebbp, CREB-binding protein) (*Cd4-Cre*), were unable to differentiate into either effector or memory CD8^+^ T cells in response to infection with *L. monocytogenes* in mice, suggesting a critical requirement of CBP in conventional effector and memory T cell generation [97]. Interestingly, another study showed that deletion of Tet2 (*Cd4-Cre*), which is responsible for erasing pre-existing DNA methylation via oxidizing 5-methylcytosine to 5-hydroxymethylcytosine [25], leads to enhanced differentiation of CD8^+^ memory T cells after LCMV infection via increased DNA methylation at *Tbx21, Prdm1, Irf4*, and *Runx3* that favored CD8^+^ effector T cell differentiation over memory T cell formation [98]. Another report contradicts the bifurcation model of T cell differentiation (naïve→ effector or naïve→ memory), instead supporting the linear model of differentiation (naïve→ effector→ memory) [99]. Using an acute LCMV infection model, this study showed that the cells that ultimately became memory T cells initially acquired a de novo methylation pattern, leading to repression of naïve T cell associated genes, as well as demethylation and expression of effector genes (such as the *Ifng, Prf1*, and *Gzmb* genes) [16,99]. It is also important to note that a subset of the methylated genes in naïve T cells eventually became demethylated and re-expressed in memory cells. This process was facilitated by loss of Dnmt3a (using the effector CD8 T cell-specific *Gzmb–Cre*) [99]. Another study showed similar outcomes, where conditional deletion of Dnmt3a (both *dLck-Cre* and *Cd4-Cre*) results in generation of more memory CD8^+^ T cells following an anti-viral (LCMV) response [100]. Other results supporting a linear model of CD4^+^ T memory cell generation were observed in both humans and mice [101]. The authors correlate DNA methylation patterns during memory T cell differentiation with gene expression and identified FOXP1 as the master downstream target of this process [101]. They showed that TF FOXP1 acts to maintain the naïve state in CD4^+^ T cells, and in mice, loss of Foxp1 (*Cd4-Cre*) leads to depletion of naïve CD4^+^ T cells [101]. Using human CD4^+^ T cells, they found that FOXP1 protein expression was highest in naïve T cells and decreased subsequently (naïve > effector > memory) [101]. The authors showed that *FOXP1* is epigenetically controlled both in the mouse and human, and the *FOXP1* locus is associated with strong progressive gain of methylation patterns with differentiation.

One study explored the role of the histone methyltransferase Suv39h1 in CD8^+^ memory T cell generation [102]. Single-cell RNA sequencing (scRNA-seq) identified that Suv39h1-depleted (germline deleted) CD8^+^ T effector cells have a defect in silencing of stem/memory genes in mice after *Listeria monocytogenes* infection, leading to increased survival and long-term memory reprogramming capacity in the effector cells [102]. From this report, it is clear that Suv39h1-mediated H3K9me3 deposition creates an epigenetic barrier on the stem/memory gene expression program in the effector cells and thus regulates the fate of T cell differentiation [102]. To better understand the CD8^+^ T cell fate decision following activation, another study was performed on antigen-specific CD8^+^ T cells during an acute infection with LCMV [103]. scRNA-seq revealed that short-lived effector cells (SLECs) lose accessibility at Tcf7 (Tcf1)- and Tcf3-occupied enhancers, which regulate genes critical for memory T cell differentiation, and these cells are therefore unable to form long-lived memory T cells [103]. On the other hand, memory precursor effector cells (MPECs) sustain chromatin accessibility at enhancers, which are highly enriched for Tcf3 (E2a) binding sites and are important for memory-related gene expression [103]. Another study showed that BH3-only, pro-apoptotic Bcl-2 family member, Bim (Bcl2l11) levels determine T effector memory (T_EM_) vs. T central memory (T_CM_) cell fate [104]. Although Bim is a pro-apoptotic protein, this report showed that Bim is highly expressed in pre-memory cells and Bim expression is negatively correlated with DNA methylation at the Bim promoter [104]. Using bisulfite sequencing on T_EM_ and T_CM_ cells sorted from C57BL/6 mice, the authors found that the CpG sites of T_EM_ cells were highly methylated compared to T_CM_ cells, which express higher levels of Bim and have low levels of DNA methylation [104]. They further showed that the TCR signal strength promotes Bim and Nur77 expression to drive more pre-memory cell generation whereas Bcl-2 antagonizes Bim in T_CM_ cells to promote memory T cell survival [104]. Another report showed that Usp16 (ubiquitin carboxyl-terminal hydrolase 16) (*Cd4-Cre*) deleted mice have reduced peripheral memory T cells and dysregulated T cell maintenance [105]. Mechanistically, they found that Usp16-mediated de-ubiquitination of calcineurin A, which is a calcium-activated phosphatase that dephosphorylates members of the nuclear factor of activated T cells (NFAT) family, leads to transcription of NFAT-targeted genes, which helps in the maintenance and proliferation of peripheral T cells [105].

A very recent study showed that ketogenesis-derived β-hydroxybutyrate epigenetically regulates CD8^+^ memory T cell development [106]. In this study, the authors documented that β-hydroxybutyrate is present in CD8^+^ memory T cells. They also showed that, β-hydroxybutyrate upregulates the expression of Foxo1 and Ppargc1a (which encodes Pgc-1α) via modification of Lys 9 of histone H3 (H3K9) at these genes with β-hydroxybutyrylation. Foxo1 and Pgc-1α cooperatively upregulate the expression of phosphoenolpyruvate carboxykinase (Pck1), which maintains the carbon flow along the gluconeogenic pathway to glycogen and the pentose phosphate pathway as well as the maintenance and formation of CD8^+^ memory T cells [106]. These results linked epigenetic modification to a metabolic pathway and CD8^+^ memory T cell development in a precise way, suggesting that the role of metabolism in epigenetic modifications should be included in future studies of T cell development.

### 2.5. Epigenetic Modulation of Natural Killer T Cells

NKT cells are regulatory T cells that serve as a bridge between the innate and adaptive immune systems [107]. In recent years, several studies have shown that NKT cell development is regulated by epigenetic modifications [20]. One study showed that depletion of histone acetyltransferase Gcn5 (*Lck-Cre*) leads to a block in iNKT cell development [104]. Mechanistically, they showed that Gcn5 pharmacological inhibition or deletion results in inhibition of the transcription of *Egr2* (early growth-responsive gene 2), which is required for iNKT cell development, since Gcn5-derived acetylation positively regulated *Egr2* transcription [58]. They further showed that Gcn5 suppression specifically represses the expression of Egr2 target genes including *Plzf*, *Runx1*, *IL2rb*, and *T-bet (Tbx21)* as Egr2 binding to the promoters of these genes is diminished [58].

Other than acetylation, histone methylation also modulates NKT cell development and function. One study showed that the promoter of PLZF (encoded by *Zbtb16*), a transcription factor critical for NKT development, exhibits a bivalent chromatin state identified by positive H3K4me3 and negative H3K27me3 modifications [50]. The authors reported that, Ezh2 depletion (*Cd4-Cre*) leads to ablation of H3K27me3 at the *Zbtb16* promoter, creating accumulation of iNKT-like CD4^+^ cells and their development into NKT2 cells whereas stabilization of H3K27me3 accounts for a significant reduction of the iNKT cell population [50]. They further showed that deficiency of the H3K27me3 demethylases Jmjd3 and Utx results in reduced expression of PLZF and reduced numbers of iNKT cells, suggesting these proteins are critical for iNKT cell generation [50]. Additional studies confirmed these observations [51,52,108]. One group provided detailed insights of Utx-mediated regulation of iNKT cell development [51]. They showed that Utx-ablated (*Vav-Cre*) iNKT cells have an increase in repressive H3K27me3 marks and a decrease in activation-H3K4me3 marks at the promoters of iNKT cell signature genes occupied by Utx [51]. They further showed that JunB regulates iNKT cell development and that Utx regulates the expression of both PLZF and JunB target genes [51]. Finally, they identified iNKT cell super-enhancers and demonstrated that Utx regulates the accessibility of super-enhancers, which play a major role in iNKT cell lineage commitment [51].

## 3. Epigenetic Modification and Cancer

Several studies have shown that aberrant epigenetic modifications of T cells are associated with different cancers. LSD1 demethylates H3K4 and H3K79 and its expression is upregulated in many cancers including T cell acute lymphoblastic leukemia (T-ALL), breast, prostate, hepatocellular, and several others [109,110]. LSD1 inhibitors are currently under clinical trials for cancer treatment [111]. Recently, one study showed that in the case of triple-negative breast cancer (TNBC), administration of LSD1 inhibitor increases CD8^+^ cytotoxic T cell trafficking towards tumor sites with increased chemokine expression including CCL5, CXCL9, and CXCL10, which are known to recruit CD8^+^ T cells to tumors (Table 1) [62,112]. They further showed that LSD1 inhibitor treatment increases the active histone mark, H3K4me2, in the promoter regions of these chemokine genes, leading to more CD8^+^ T cell recruitment and disease remission [62]. Another study using a mouse breast cancer model showed that inhibition of nuclear Lsd1 (nLsd1) promotes infiltration of IFN-γ/TNF-α-expressing CD8^+^ T cells into tumors [63]. They also found, in dysfunctional “exhausted” CD8^+^ T cells, that the transcription factor Eomes and nLsd1 are co-expressed and that Lsd1 promotes T cell exhaustion by bivalent post-translational modification at lysine 641 of Eomes to prevent its nuclear entry [63]. Therefore, targeting nLSD1 may prevent T cell exhaustion and yield better therapeutic potential against tumors. Similar anti-tumor effects were obtained from a mouse melanoma study by using Lsd1 inhibitor or deleting Lsd1 (CRISPR/Cas9-mediated gene deletion) [64]. They found that Lsd1 ablation leads to activation of type 1 interferons (IFNs) and significant increases in both CD4^+^ and CD8^+^ T cells in melanoma tumors [64]. Mechanistically, they showed that ablation of Lsd1 leads to upregulation of ERV (endogenous retroviral element) transcription, resulting in activation of type-1 IFNs [64]. Taken together, these results clearly demonstrate that LSD1 plays an important role in effector T cell function, but its role in normal T cell development and differentiation is yet to be determined.

One report showed that treatment with the DNA hypomethylating agent (HMA), decitabine (DAC), leads to increased CD8^+^ T cell tumor infiltration and inhibits tumor growth via CD8^+^ T cell-dependent activity in different mouse tumor models [113]. Using healthy human donors, the authors further showed that DAC treatment leads to T cell activation and expansion of the granzyme B^high^, perforin^high^ effector subpopulation and also increased expression of NFATc1/A, both of which contribute to the cytolytic activity of CD8^+^ T cells [113]. Collectively, this study identified that DNA methylation suppresses a subset of genes required for CD8^+^ T cell activation and that HMA treatment reprograms these genes to become activated, promoting cytolytic activity. 

Exhausted CD8^+^ T cells (T_ex_) have limited function as effector cells against chronic infections and cancer due to extensive transcriptional changes and increased co-expression of inhibitory receptors [17,18]. Recently, one study reported that deletion of the HMG-box transcription factor Tox (*Cd4-Cre*) leads to complete ablation of T_ex_ formation (Table 1) [65]. Mechanistically, they showed that depletion of Tox leads to increased chromatin accessibility at genes linked to terminal CD8^+^ T effector cell differentiation, including *Klrg1*, *Zeb2*, *Gzma*, *Gzmb*, etc. They also showed that Tox directly binds with the acetyl transferase Kat7 as well as repressive epigenetic regulators (e.g., Dnmt1, Leo1), suggesting that Tox interacts with proteins involved in both the opening and closing of chromatin, leading to gene regulation [65]. Similar observations were made by another study that found that Tox and Tox2 cooperate with Nr4a (Nuclear Receptor Subfamily 4 Group A Member 1) transcription factors to promote CD8^+^ T cell exhaustion [66]. Finally, another group identified Tox as a critical modulator for the differentiation of tumor-specific T (TST) cells in a mouse model of liver cancer [67]. TST is a distinct state compared to functional effector or memory T cell states [114]. TSTs, which are mainly found in solid tumors, are dysfunctional as tumors progress despite their presence [115]. The authors showed that, in dysfunctional TST cells from tumors and in exhausted T cells obtained from chronic viral infection, Tox levels are significantly elevated [67]. Ablation of *Tox* (*Lck-Cre*) in TST cells in tumors diminished T cell exhaustion via downregulation of inhibitory receptor genes including *Pdcd1*, *Tigit*, *Cd244*, *Entpd1*, and *Havcr2* through inaccessible chromatin [67]. Although Tox deletion abrogates T cell exhaustion, TST cells still remained dysfunctional, suggesting additional molecular mechanisms for exhaustion still remain to be identified [67]. Collectively, these studies shed light on CD8^+^ T cell exhaustion and establish the importance of Tox-mediated epigenetic modifications in this process. These findings also suggest that Tox may be a therapeutic target to decrease exhaustion of CD8^+^ T effector cells.

As mentioned previously, epigenetic dysregulation has also been correlated with autoimmune disorders. Although beyond the scope of this review, readers are referred to [25,26], which address this subject.

## 4. Perspective and Future Directions

Recent studies have shed light on the importance of epigenetic regulation in the T cell differentiation process. Several mutations in epigenetic modifiers have been reported, which contribute to disease pathogenesis due to abnormal changes in gene expression required for T cell differentiation [116]. Specific inhibitors of chromatin remodeling proteins are currently under clinical trials, which may have novel treatment options for patients. It is also important to note that, as discussed in this review, some inhibitors are associated with unwanted toxicity or risk of autoimmune diseases. At the same time, various studies point out the rate-limiting factors that activate or suppress CTL functions. These findings may help identify novel approaches to improve immunotherapy for various cancers. Therefore, continuous efforts to understand the T cell differentiation process and the epigenetic regulators of this process will yield valuable information, which may be used for translational purposes.

## Figures and Tables

**Table 1:** Major epigenetic regulators associated with T cell differentiation.

Regulator	Cell Types	Modification/Function	References
Dot1L	Th-2, CD8^+^	H3K79me2	[36,37]
Menin	Th-2	Menin/TrxG complex promotes H3K4me3	[38,39,40,41,42]
Cxxc1	Th-1, Th-2, Th-17	Cxxc1/TrxG complex inhibits/promotes H3K4me3 in gene specific manner	[43,44,45,46]
ATF7ip	Th-17	ATF7ip/SETDB1 complex promotes H3K9me3	[47,48]
Utx	Th-17, iNKT	Histone demethylase	[49,50,51,52]
Hdac3	CD4^+^, CD8^+^, iNKT	Histone deacetylation	[53,54,55]
Tcf1/ Lef1	CD4^+^, CD8^+^	Histone deacetylation	[56]
Gcn5	Th-1, Th-17, iNKT	Histone acetylation	[57,58]
Rcor1	Treg	Rcor1 is a part of CoREST complex which mediates histone deacetylation	[59,60]
Ezh2	CD8^+^, iNKT	H3K27me3	[37,50,61]
Lsd1	CD8^+^ CTLs	H3K4 and H3K79 demethylase	[62,63,64]
Tox	Exhausted CD8^+^	Binds to epigenetic regulators to change gene expression	[65,66,67]

## Data Availability

Not applicable.
